# From Farm to Fable

**DOI:** 10.3201/eid2204.AC2204

**Published:** 2016-04

**Authors:** Byron Breedlove

**Affiliations:** Centers for Disease Control and Prevention, Atlanta, Georgia, USA

**Keywords:** art science connection, emerging infectious diseases, art and medicine, about the cover, Spring in the Country, From Farm to Fable, infectious diseases, Grant Wood, regionalism, foodborne diseases, food safety, public health

**Figure:**
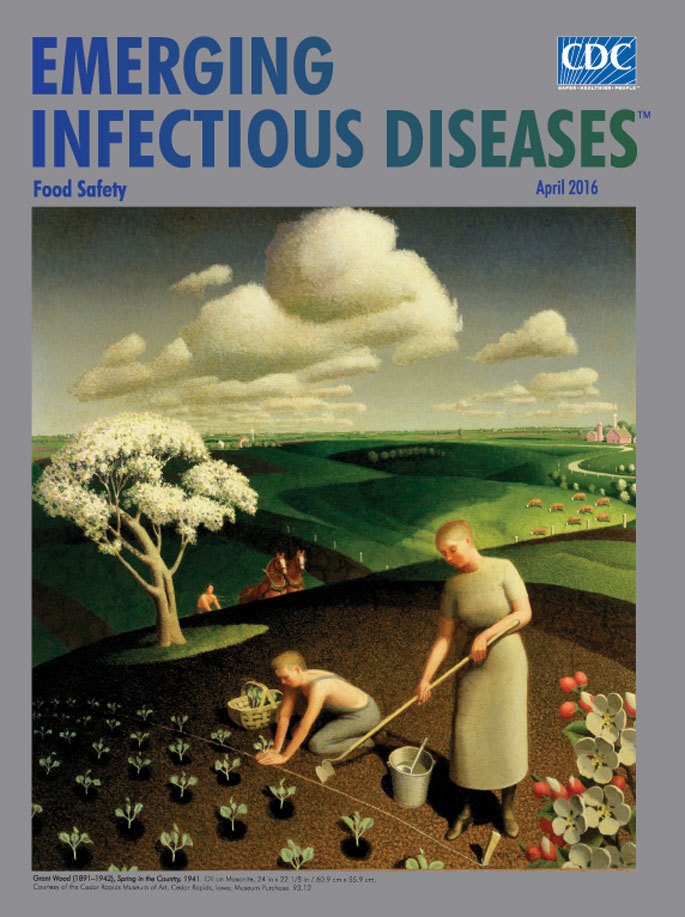
**Grant Wood (1891–1942), *Spring in the Country*, 1941. Oil on masonite, 24 in × 22 1/8 in / 60.9 cm × 55.9 cm.** Courtesy of the Cedar Rapids Museum of Art, Cedar Rapids, Iowa; Museum Purchase. 93.12

Grant Wood once quipped that “All the good ideas I ever had came to me while I was milking a cow.” Though spurious, his comment underscores how the memories of farm life from his youth provided substance and inspiration for much of his artwork, particularly his landscapes. Wood was in fact born on a farm near Anamosa, Iowa, in 1891, and he lived there until his father died in 1901. His family moved to Cedar Rapids, Iowa, soon thereafter, when Wood was 10 years old.

In this month’s cover image, Wood’s *Spring in the Country,* a rolling patchwork of resplendent green Iowa farmland recedes to the distant horizon. Soaring clouds hover above the fields, swelling from the horizon till they meet the sky in the top third of the painting. As a plainly dressed woman prepares the soft ground with a hoe, a young man in overalls carefully takes another seedling from the basket next to him and presses it into its chosen spot. A ladle floats in the bucket of water, within easy reach for watering the young plants. Near a flowering tree, cresting a hill, a farmer relies on a pair of farm horses for tilling his fields on this warm early spring day. Also visible across this pristine panorama are more flowering trees, a small herd of white-faced cattle, an orderly row of fence posts, and other farms. These 3 people approach their day’s labor with a relaxed ease.

One of the last works Wood completed before dying of pancreatic cancer hours from his 51st birthday, *Spring in the Country*, shares motifs with many of his other landscape paintings. It offers viewers a peaceful respite in this lush green, rolling farmland, a place free from signs of modernization or industry. James Dennis characterized those works as Wood’s “idealized, fantasy Iowascapes.” A second look confirms that power lines and telephone poles do not transect the landscape; no tractors, combines, or threshers thrum anywhere in this vast, pastoral countryside.

Notes from the University of Virginia American Studies Program state that “Although Wood's landscapes were received as idealized visions of America's heartland, a critical feature of the paintings is that they are pointedly not contemporary. America in the 1930s was in the grips of the machine age, erecting skyscrapers and building technological marvels like the Hoover Dam—yet machines do not enter into Wood’s landscapes.” Nor does Wood depict the sort of desperate toil and plight that would have been characteristic of the Great Depression from which the world was emerging.

While Wood was painting his final homages to the bygone simple farm life of his youth, agriculture was in the throes of major changes. Following a series of mechanical refinements during the 1930s that resulted in greater speed, power, and comfort, tractors began displacing farm horses and mules, though not outnumbering them until the early 1950s. Having reliable mechanical power in turn gave rise to related innovations and modifications to farm implements, enabling farmers to work faster and to farm more land.

Agriculture-related innovation during that time also led to the development of new varieties of crops and livestock, more effective pesticides, improved fertilizers, and better irrigation techniques. Among the dynamic, global forces that accelerated changes in food production and farming were the burgeoning expansion of mass transportation, shifts in population from rural to urban areas, and, of course, the demands and difficulties that resulted from World War II.^1^ What were once local or regional enterprises now operated on national and global scales.

Farming became more specialized, and fewer commodities were grown and sold per farm. The pattern of ever more efficient food production and the advent of larger-scaled food processing and distribution has increased the risk and the scale of outbreaks of foodborne disease.

Though some foodborne diseases that were common near the start of the 20th century—during the time Wood was living on his family’s farm—are now rare, others persist. Today there are also newly recognized and emerging pathogens, new toxins, and increasing problems with antibiotic resistance, leading to estimates from the World Health Organization that unsafe food is linked to the deaths of an estimated 2 million people annually from more than 200 food-related diseases.

Preventing, tracking, and investigating foodborne illnesses is an increasingly complex public health endeavor. It involves wide-reaching collaboration among agriculture and food processing industries, regulatory and public health agencies, and consumers, and its range can potentially be global. It is jolting to realize that Wood’s nostalgic visual fables of farm life, with their white-faced cattle and farm horses, rolling green farm lands, and, country folks working under a warm spring sky, would in reality harbor many of the more than 250 bacterial, viral, and parasitic pathogens that today are known to cause foodborne illness.

## Footnotes

1 Researcher Katherine J. Curtis White cited a body of “literature investigating agricultural economics and population dynamics focuses on the ‘pre-mechanization’ period and the ‘post-mechanization’ period, with 1940 marking the decade of transition.”
